# Factors contributing to online child sexual abuse in Bangladesh: A qualitative inquiry

**DOI:** 10.12688/f1000research.141568.2

**Published:** 2024-01-16

**Authors:** Md Redwanul Islam, Muhammad Ibrahim Ibne Towhid, Merium Salwa, Anika Tasnim, Wai Wai Mroy, Md Maruf Haque Khan, Md Atiqul Haque

**Affiliations:** 1 Department of Public Health and Informatics, Bangabandhu Sheikh Mujib Medical University, Dhaka, 1000, Bangladesh; 2 Department of Palliative Medicine, Dhaka Medical College Hospital, Dhaka, 1000, Bangladesh

**Keywords:** Online Child Sexual Abuse; Root Cause; Health Problem; Children; Qualitative study; Bangladesh

## Abstract

**Background:**

Children globally, including in Bangladesh, are facing various forms of online sexual abuse including sextortion, exploitation, body shaming, and blackmail. They are also coerced into engaging in intimate activities, harassed through the sending of sexual content, among other forms of abuse. We aimed to explore the root cause of online child sexual abuse (OCSA) in Bangladesh.

**Methods:**

This qualitative research design utilized in-depth interviews (IDIs) and key informant interviews (KIIs) between February and April 2022. The study sample comprised 21 school-going children aged 13−17 years, selected from two different geographical settings (10 from rural areas and 11 from urban areas) in Bangladesh using purposive sampling techniques. They participated in in-depth interviews (IDIs) while additional data was obtained through key informant interviews (KIIs) with 11 multidisciplinary stakeholders.

**Results:**

Children from both rural and urban areas reported facing abuse in various ways, such as being asked to send naked photos, being invited to be naked in video calls, and being invited to have virtual sex, among others, over the internet.

**Conclusions:**

The government should consider integrating OCSA education into secondary levels. Additionally, there should be efforts to ensure a safe online environment through content scrutiny, promotion of outdoor activities, and community campaigns. An anonymous reporting system must be implemented, and strict measures under the Children's Act must be enforced against perpetrators. Further intervention studies are needed to ensure effective child protection measures in Bangladesh.

## Introduction

The internet makes it possible for people to interact and communicate with one another instantly ( Sethi and Ghatak, 2018). According to the Digital Global Report ( Kemp, 2022a), 62.5% of people worldwide have internet access, and social media users comprise 58% of the world population. Globally, it is estimated that 75% of children aged 12−17 years use the internet regularly, mainly for education and entertainment purposes. Yet, despite its many beneficial roles, access to the internet exposes children to the risk of abuse, such as stalking, cyberbullying, blackmail, and sexual exploitation ( UNICEF, 2022). According to a multi-country study conducted by UNICEF, 80% of children are exposed to online sexual abuse and cyberbullying ( UNICEF, 2022).

Children experience abuse over the internet for several reasons, including inadequate parenting and lack of legal support ( NCTSN, 2009; WHO, 2022), as well as inadequate knowledge of safe internet use and online abuse of children ( OECD, 2020). However, extant research also shows that female children and those living in rural areas are comparatively more vulnerable to online sexual abuse ( Fattah and Kabir, 2013).

This is particularly relevant for Bangladesh, a total of 167.1 million residents, 60.3% live in rural areas, while 39.7% live in urban areas ( Kemp, 2022b). Among them, approximately 30% of the Bangladeshi population has internet access. Although, unexpectedly, a large number of these internet users are children, it is difficult to estimate their exact number because most children use their parents’ electronic devices to access online content ( Chahal *et al.*, 2013).

A UNICEF survey conducted in 2019 showed that around 32% of children aged 10−17 years in Bangladesh face cyberbullying and cyber harassment ( Nabi, 2019; UNICEF, 2019). More recently, Mubassara *et al.* (2021) revealed that around 21% of rural children in Bangladesh received sexually explicit messages, while 17% received videos or pictures with sexual content. Available evidence further indicates that 56% of male and 64% of female children in Bangladesh face online sexual abuse ( Ain o Salish Kendra, 2023).

Thus, online child sexual abuse (OCSA) is a growing problem in Bangladesh, but this issue is highly prevalent in other countries. Still, due to the paucity of studies focusing on the quantitative aspect of this problem ( Shamim, 2017), evidence on the OCSA causes is limited. To address this gap in pertinent literature, in the present study, we aimed to identify the root causes of OCSA in Bangladesh.

### Conceptual framework

The following theories constitute the theoretical framework to comprehend online child sexual abuse: the ecological model, the pathways model of child sexual abuse ( Krug *et al.*, 2002; Ward and Siegert, 2002).

Belesky’s socio-ecological model explains the risk factors of child abuse, which are divided into four interrelated and mutually embedded levels: (a) individual, (b) relationship, (c) community, and (d) societal ( Krug *et al.*, 2002). This model may provide insight into the causes of OCSA.

The individual level focuses on personal factors such as personal history, behavior, and attitudes that increase the risk of becoming a victim or perpetrator. The relationship level refers to the influence of relationships with parents, partners, family members, and peers on behavior. The community level considers violence that occurs in schools, workplaces, and neighborhoods that increases the risk of becoming a victim. The societal level refers to the impact of social norms, values, and economic, political factors on society. Overall, the model identifies the process of violence from an individual level to family, community, and societal levels.

The pathways model of child sexual abuse proposed by Ward and Siegert (2002) partially explains the cause of online child sexual abuse in Bangladesh. The model encompasses five etiological pathways including intimacy deficits, deviant sexual scripts, emotional dysregulation, antisocial cognitions, and multiple dysfunctional mechanisms to explain possible offensive sexual behaviour. These pathways describe the combined relationship among emotional, psychological, and other mechanisms for understanding the preconditions of child sexual abuse. The first pathway suggests that offenders substitute children for adults due to intimacy deficits and self-esteem issues. The second pathway suggests that individuals with distorted sexual scripts may have dysfunctional relationship schemas that contribute to premature sexualization of children. The third pathway suggests that individuals with poor emotional regulation may use sex as a way to cope with their emotions. The fourth pathway suggests that patriarchal attitudes may contribute to child-adult sexual relationships based on the superiority of men in society. The fifth pathway involves pedophilic behaviors, where adults have an interest in engaging in sexual acts with children.

## Methods

### Study design

The data for this qualitative study was obtained from 32 respondents, 11 of whom took part in key-informant interviews (KIIs) and 21 participated in in-depth interviews (IDIs). The demographic characteristics of the interviewees are presented below in Table 1. IDIs were conducted face-to-face with school-going children aged 13−17 in isolated places at their households, whereas KIIs focused on multidisciplinary professionals (child psychiatrist, psychologist, child rights activist, sociologist, lawyer, NGO professional, and Information and Communication Technology (ICT) teacher) and were conducted online via the Zoom platform. Due to the COVID-19 pandemic, participants were purposefully selected to ensure representation from rural and urban areas while maintaining homogeneity.

**Table 1.** Demographic characteristics of the interviewees.

Participants	Age	Gender	Designation/Level	Profession
KII-01	30-40	Female	Child Protection Officer	NGO Professional
KII-02	30-40	Female	Senior Officer	NGO Professional
KII-03	50-60	Male	Professor	Psychologist
KII-04	30-40	Male	Senior Officer	NGO Professional
KII-05	50-60	Male	Professor	Lawyer
KII-06	40-50	Male	Assistant Professor	ICT Teacher
KII-07	50-60	Male	Associate Professor	Child Psychiatrist
KII-08	40-50	Female	Assistant Professor	ICT Teacher
KII-09	60-70	Female	Professor	Child Right Activist
KII-10	30-40	Female	Assistant Professor	ICT Teacher
KII-11	50-60	Female	Professor	Sociologist
IDI-01	13-17	Female	Secondary	Student
IDI-02	13-17	Male	Secondary	Student
IDI-03	13-17	Female	Secondary	Student
IDI-04	13-17	Female	Secondary	Student
IDI-05	13-17	Female	Secondary	Student
IDI-06	13-17	Female	Secondary	Student
IDI-07	13-17	Male	Secondary	Student
IDI-08	13-17	Male	Secondary	Student
IDI-09	13-17	Male	Secondary	Student
IDI-10	13-17	Male	Secondary	Student
IDI-11	13-17	Male	Secondary	Student
IDI-12	13-17	Male	Secondary	Student
IDI-13	13-17	Male	Secondary	Student
IDI-14	13-17	Male	Secondary	Student
IDI-15	13-17	Male	Secondary	Student
IDI-16	13-17	Male	Secondary	Student
IDI-17	13-17	Female	Secondary	Student
IDI-18	13-17	Female	Secondary	Student
IDI-19	13-17	Male	Secondary	Student
IDI-20	13-17	Female	Secondary	Student
IDI-21	13-17	Female	Secondary	Student

IDI: In depth interview, KII: Key informant interview, ICT: Information communication technology, NGO: Non-governmental organization.

### Study setting and duration

To reach out to both urban and rural children for IDIs, we purposefully selected Kadomtoli Thana (sub-district) of Dhaka as an urban area and Kamalgonj sub-district of Moulovibazar as a rural area. The study was conducted between February and April 2022.

### Study procedure

The interview guides for KIIs and IDIs were designed based on three thematic areas: defining the problem, the causes behind the problem, and possible preventive strategies. A consultative meeting was held at the Department of Public Health and Informatics at Bangabandhu Sheikh Mujib Medical University (BSMMU) with public health experts, anthropologists, lawyers, child health specialists, and sociologists to outline the methodology and finalize the interview guides. The research team was comprised of experts from different academic backgrounds and research institutions, with credentials including PhD, MD, MBSS, MSS and LLB, which helped us investigate the issue from various angles. Pre-test interviews were conducted to finalize the interview guides, and the data from the pre-test interviews were excluded from the analysis.

Interviews were conducted by the first author and three co-authors (M.R.I., A.T., M.I.I.T., and M.S.) including two males and two females, were engaged in all the interviews. Female children were interviewed by female researchers. The researchers introduced themselves to the interviewees to establish rapport and provide a brief overview of the study before commencing the interviews. The researchers also took into consideration potential biases and assumptions during the interviews.

The duration of interviews was 15 to 30 minutes for individual IDIs and 30 minutes to one hour for KIIs. Interviews were recorded with proper consent and transcribed verbatim immediately after the interview. Non-verbal expressions were also noted. The interviews were conducted until new information was obtained from the respondents. Once we observed repetition of information, we concluded the data collection process.

### Data analysis procedure

The interview transcripts were analyzed manually using qualitative content analysis methodologies, focusing on both the manifest and latent contents. To maintain a strong connection between the transcriptions and the conducted interviews, the interviewers transcribed the audio recordings verbatim into Bengali, the spoken language of Bangladesh. Three authors (M.R.I., A.T., and M.S.) simultaneously read the transcripts, reviewed them, and confirmed their accuracy for analysis. They were involved in the analysis process, firstly meaningful phrases relevant to the objective of the study were selected from the text as meaning units. In the subsequent steps, all condensed meaning units were coded and grouped into categories ( Elliott, 2018), allowing an overarching theme to emerge. The depth and level of abstraction of both the manifest and latent content were open to interpretation ( Graneheim and Lundman, 2004). While the analysis of latent content concentrated on revealing hidden meanings, the interpretation of manifest content tried to characterize the text’s evident and readily apparent aspects.

### Ethical considerations

Ethical permission for conducting this study was granted by the Institutional Review Board of BSMMU, Dhaka (Memo no. BSMMU 2022/33115). The ethical approval and the purpose of this study has been clearly stated to the interviewees. Written permission was obtained from guardians to conduct interviews with children, and assent from the children was also obtained. In collecting data on children’ experiences of online sexual abuse, the safety and sensitivity of all youth participants were taken into account during the interviews and throughout the entire research process. Prior to commencing each interview, children were informed that they could terminate the interview at any time and skip any questions they did not wish to answer. Their parents were fully informed about the focus of this study on child abuse and the inclusion of emotionally charged questions that their children can find distressing. They were also advised that their children could opt out of participating in the interview at any time. Nonetheless, to avoid upsetting or re-traumatizing the participants, interviewers did not directly ask them about their own experiences of online sexual abuse. In addition, participating children were encouraged to seek support from a trusted adult if they felt uncomfortable during the interview, and the interviewers completed each interview by asking how participants felt. However, none of them were uncomfortable after completing the interview.

### Operational definitions

**Child.** We considered a child as a person aged up to 18 years, adopting the same definition of a child as outlined in the United Nations Convention on the Rights of the Child.

**Youth.** In Bangladesh, the definition of youth in Bangladesh varies. According to the National Youth Policy (2017), individuals aged between 18 and 35 are considered youth, while the Bangladesh Bureau of Statistics (2017) defines youth as those aged between 15 and 29.

**Online abuse.** Online abuse is regarded as an extension of traditional forms of abuse that takes place in cyberspace, allowing perpetrators to hide behind screens. It involves willful and repetitive behaviors that have psychological consequences ( Agatston *et al.*, 2007) and serve as a means to express social aggression through electronic communication devices ( Çetin *et al.*, 2011). This term encompasses various forms of abusive interpersonal behaviors, including online bullying, stalking, sexual solicitation, and problematic exposure to pornography ( Mishna *et al.*, 2014).

**Online sexual abuse.** It includes two separate expressions as sexual solicitation and sexual interaction. Receiving a request from a perpetrator to provide sexual information or engage in sexual activities is referred to as sexual solicitation, whereas sexual contact is defined as actively engaging in sexual actions online ( Baumgartner *et al.*, 2010).

**Cyberbullying.** Cyberbullying, also known as electronic bullying or online social cruelty ( Agatston *et al.*, 2007), includes ‘willful and repeated harm inflicted’ ( Hinduja and Patchin, 2008) toward another. Cyberbullying is unique in its use of electronic communication technology as the means through which to threaten, harass, embarrass, or socially exclude ( Hinduja and Patchin, 2008; Cook *et al.*, 2007; Patchin and Hinduja, 2006). Cyberbullying (CB) is a form of interpersonal aggression that occurs through electronic means, such as texting, online chats, or social media websites ( Olenik-Shemesh *et al.*, 2017).

**Sexting.** Sexting is sending, receiving, or forwarding sexually explicit messages, photographs, or images, primarily between mobile phones, of one to others. It may also include the use of a computer or any digital device ( Van Ouytsel *et al.*, 2017).

## Results

**Figure 1.**
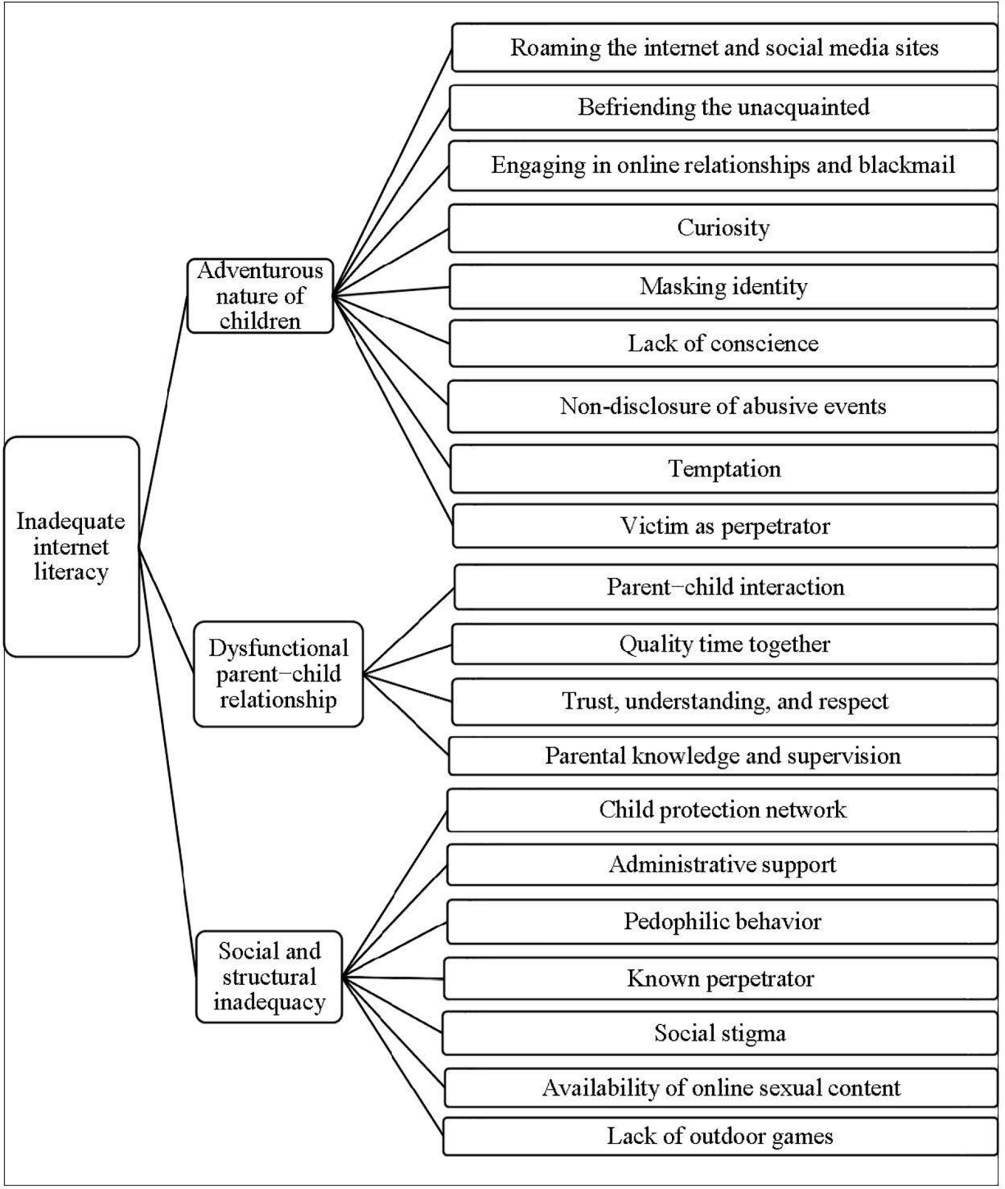
Decision tree of the root cause of online child sexual abuse.

The main theme that emerged from the analysis is inadequate internet literacy as the root cause of online child sexual abuse (OCSA) in Bangladesh, which is also reflected in the categories. The following three categories resulted from the analysis: (a) the adventurous nature of children, (b) dysfunctional parent−child relationship, and (c) social and structural inadequacy ( Figure 1). As each subcategory helps to establish the causes of OCSA, they shed light on how OCSA should be viewed from a socio-cultural point of view. Each category consists of 4 to 9 subcategories.

### Adventurous nature of children

1. ***Roaming the internet and social media sites***

The availability of internet-enabled devices, such as smartphones, tablets, laptops, etc., encourages children in both rural and urban areas that have internet access to spend more time online. They explore different social media sites and, as they do not know who is on the other side of the internet, they can easily fall into the abuser’s trap. According to a female respondent:

*“I have many friends on Facebook, but some have bad motives and can do the worst things. My friend shared her personal information with an unknown person and her Facebook account was hacked. Afterward, the hacker irritated her by asking for more nude images or else the hacker would make her photos viral.”*

     -(IDI-18, Grade 9)

2. ***Befriending the unacquainted***

As children may utilize different social media platforms such as Facebook, Messenger, WhatsApp, Instagram, Snapchat, Viber, etc., this can have both positive and negative impacts on their lives ( Soniya, 2017). While one of the known risks of such online presence is excessive use of electronic devices, internet use also allows perpetrators to collect children’s contact details from the recharge shops and attempt to befriend them online. They would subsequently irritate children through online audio and video calls. On this issue, a child psychiatrist noted:

*“The perpetrator gets close to a child by posing as a good friend and gradually starts abusing the child. Perpetrators often collect victims’ contact numbers from recharge shops and search for them on Imo, WhatsApp, and other social media. Children, without understanding the consequences, chat out of curiosity.”*

     -(KII-07, Child Psychiatrist)

3. ***Engaging in online relationships and blackmail***

Children who engage in romantic relationships through the internet are at risk of becoming victims of sexual abuse, as perpetrators who have developed an online relationship with them may pressure them to share nude photos or even insist on physical relationships, as stated by an ICT expert.

Even if a youth shares personal documents just once, there is a risk of being abused, given that perpetrators may threaten to publish private relationships in front of family members, classmates, society, etc., if the victim refuses to comply with their requests. When in love, children may sometimes share their personal/intimate photos and videos with their romantic partners online, without realizing that these can be later used for blackmail. As noted by an informant:

*“I became engaged in a love affair with one of my classmates. Due to the pandemic, our love affair continued online. As time went by, he started asking me to share nude images. I refused to share any images online, but eventually, he started blackmailing me. He threatened that he would inform my family members about our love affair. He said that he would present me in a negative way to my family. There were only two choices left: listen to him or commit suicide.”*

     -(IDI-18, Grade 9)

According to the respondents, when a child becomes a victim of abuse, they are often blamed for the abuse rather than being supported, as people tend to think that the victim must have done something wrong to provoke the abuse. This was aptly surmised by a rural Grade 9 female informant who stated:

*“I kept my relationship hidden from my family members because my family is conservative and would take away my mobile phone if they knew I used it for social media access. After a few months, we broke up as the relationship was no longer working. But then, my (ex-boyfriend) started blackmailing me. As a result, I started experiencing deep depression. The family members came to know some of the details, and they accused me. They cursed me and said I should die to avoid causing further damage to their reputation and honor.”*

     -(IDI-18, Grade 9)

4. ***Curiosity***

Easy internet accessibility increases children’s curiosity to explore new things online, but it also elevates the risk that they may become involved in harmful activities. According to one of the interviewed:

*“Children can easily buy an internet package for unlimited social media usage with as little as 10 Bangladeshi taka (equivalent to 0.1 USD). These unlimited internet packages come with a limited-time offer from mobile phone operators. Children have a tendency to become involved in the internet and engage in unnecessary activities, which also leads to an addiction to viewing pornographic content and opens the door for sexual abuse.*”

     -(KII-08, ICT Teacher)

5. ***Masking identity***

Abusers’ ability to easily mask their identity increases the likelihood that children would fall into abusers’ traps or even start abusing others by modeling harmful behaviors they witness online. Many perpetrators targeting children create fake social media profiles, such as Facebook IDs, using female names and photos, and start disturbing the friends of the actual user through fake accounts. Perpetrators also blackmail the victim for money with personal photos and videos. Such proliferation of fake Facebook accounts makes it impossible for youth to verify which account is genuine. Children may also become abusers by learning from vulgar comments they read in different online Facebook groups. On this issue, one of the participating ICT teachers commented:

*“Children are creating fake social media accounts and are using them to irritate others. Yet, we have no surveillance system in our country, indicating that an urgent need to improve the intelligence software to monitor children.”*

     -(KII-08, ICT Teacher)

6. ***Lack of conscience***

Children often share personal information (including revealing images) with their romantic partners online to show off, while also seeing this as a way to gain trust and assurance of commitment in their relationship. However, most are unaware that this creates a threat of sextortion, given that the person they consider their partner may start abusing their trust and demand nude photos. It is also not uncommon for those in a relationship to give their partner access to their social media account. The difficulties that may arise as a result were outlined by a female respondent from a rural area in Grade 10 as follows:

*“My ex-boyfriend asked me to send my nude photo just once as evidence of our relationship and proof that I love him. I was so much in love with him that I sent him the picture. When we were in a relationship and while we saw each other in person, he used to check my mobile phone and get access to my Facebook ID and password. I later tried to change my ID and password, but I was unable to do so.”*

     -(IDI-21, Grade 10)

Children often fall into such traps as they do not appreciate that sharing personal information might be dangerous for them. This is particularly problematic when they are in romantic relationships, as they feel connection to their partners and see no harm in sharing inappropriate photos, videos, and other personal information with the loved one. Yet, if the relationship fails, the other person may use this personal information to threaten or humiliate them.

7. ***Non-disclosure of abusive events***

When children become victims of online sexual abuse, they rarely report these incidents due to shame and worry that people (including their parents) will blame them instead of supporting them or trying to solve the problem. The victims also remain silent out of fear that their identity will be disclosed by the perpetrator and they will be embarrassed in front of their friends, relatives, and family members. That is why most cases of OCSA remain unreported.

8. ***Temptation***

Perpetrators often tempt children to start communicating with them by offering gifts, jobs, outings, or visits to a restaurant, or enticing them in some other way children would find appealing. On this topic, one of the participating ICT teachers stated:

*“Perpetrators abuse children with power influence. If you cannot stop power influence, you cannot stop child abuse. Children with the support of their influential parents at times develop an attitude that they can do whatever they like and nobody can punish or stop them. Some parents even shelter their children from harmful activities rather than stopping them from engaging in abusive events.”*

     -(KII-06, ICT Teacher)

9. ***Victim as perpetrator***

Children who have experienced sexual abuse and were not provided adequate support are likely to start tolerating such abuse and even become perpetrators. As one of the respondents took part in our study observed:

*“When a child is sexually abused and tolerates it, there is a strong possibility that the child might abuse another child. In addition, children who see pornography want to fulfill their needs by abusing others.”*

     -(KII-07, Child Psychiatrist)

### Dysfunctional parent−child relationship

1. ***Parent−child interaction***

The professionals that served as key informants in this study concurred that, as parent−child interaction in Bangladesh is generally poor, children are at an increased risk of OCSA. Unfortunately, most parents do not find spending quality time with their children and trying to understand them very important. In addition, as the Bangladeshi society discourages discussions related to sexual events within the family, children often turn to the internet to satisfy their curiosity, which exposes them to the potential abuse. According to one of the interviewed ICT teachers:

*“Children hide the worst experiences because they are unable to discuss abusive events with their parents due to poor child−parent interaction. In some cases, children cannot tolerate the negative experience and attempt suicide.”*

     -(KII-08, ICT Teacher)

2. ***Quality time together***

Some parents spend time with their children, but this may not be a quality time, as parents rarely engage with their children as friends. This barrier in the parent−child relationship makes it difficult for children to confide in their parents and report any abusive events, although they have the urge to share. Still, as noted by an urban female participant in Grade 10, some children have positive relationships with their parents:

*“My friend was harassed by her ex-boyfriend. The boy shared my friend’s images on Facebook. I asked her to share this event with her parents, and eventually, she did. Her family supported her and also brought the situation under control.”*

     -(IDI-21, Grade 10)

3. ***Trust, understanding, and respect***

Most respondents stated that trust and respect are essential in the parent−child relationship, which is lacking in most Bangladeshi families. As a result, children do not have the opportunity to share abusive events with their parents because they do not have a trustworthy, understanding, and respectful relationship with their parents. As noted by a respondent:

*“I told my parents I was going through a traumatic situation, but they were not sympathetic. My mother even told me to commit suicide and said my abuse event was a disgrace to our family.”*

     -(IDI-18, Grade 9)

In Bangladesh, the relationship between parents and children is often strained, as parents do not understand their children, as explained by one of the respondents who participated in this study:

*“A girl called me and said she was traumatized because her boyfriend disclosed their intimate pictures on social media. Her parents came to know about it and started beating her instead of trying to understand her. She called me to seek help and see if I could control the situation.”*

     -(KII-11, Sociologist)

Yet, even when children report abusive events to their parents, they do not take steps to protect against abusers due to social shame, as explained by the child psychiatrist that served as one of our informants:

*“The parents of a female child brought her for psychological counseling. She was sexually abused online by her private tutor, which the family members later revealed. She did not initially share anything with the family members as she was not close with her parents. However, the girl’s family members found certain odd changes in the girl’s behavior. She wanted to be left alone and stopped interacting with other family members. She was disturbed all the time and even urinated in bed. The parents did not want to take any legal action against the boy. They thought taking legal action would be a hassle for the family and they would be harassed, which would damage their reputation.”*

     -(KII-07, Child Psychiatrist)

4. ***Parental knowledge and supervision***

According to our study respondents, Bangladeshi parents over the age of 40 are not well equipped to use the internet and are not accustomed to using modern technologies. As a result, they cannot even imagine that their children could be abused online. Sometimes, as parents have no knowledge of the scope of online content, they do not know which websites are helpful to their children, let alone how to prevent their children from accessing harmful websites or meeting unwanted friends, fraudsters, and perpetrators online. Consequently, they are incapable of making their children aware of safe internet use. Children are also unwilling to share their negative experiences as they believe their parents cannot protect them from something they do not understand, as explained by a key informant interviewee:

*“Parental ignorance is a cause of OCSA. Due to their lack of internet knowledge, parents cannot protect their children in terms of internet difficulties. There is some parental control software, but guardians do not know how to use it. Parents give their children smartphones but do not even care what websites they visit or who they meet online.”*

     -(KII-06, ICT Teacher)

During the initial months of the COVID-19 pandemic, all classes were conducted online, children had to use the internet to participate in school activities. Whether or not children used their own or their parents’ devices, many parents did not monitor their children’s online activities. However, it is well known that spending more time online increases the chances of interactions with unknown and anonymous users, thus exposing them to potential abuse.

### Social and structural inadequacy

1. ***Child protection network***

According to Children Act 2013 of Bangladesh, every upazila (sub-district) and even the union (lowest administrative unit) is supposed to have a child protection network. In addition, a committee comprising children, parents, teachers, and local government representatives is supposed to be formed, allowing victims to come forward to report incidents of abuse through a child representative. However, only a few such committees and networks have been established. Even when such committees exist, they do not function properly, and no child representative is available for underage victims of abuse. According to one of the NGO workers that participated in the interviews:

*“NGOs are trying to ensure that child representatives are included in the child protection committees to enhance the anonymous reporting system. However, although child protection networks were supposed to be formed at the union, upazila, and district levels, during our field visits, we found that the committees at those levels were not properly functioning.”*

     -(KII-01, NGO Professional)

This issue is further exacerbated by the fact that, even though some laws in Bangladesh directly or indirectly address protection against child abuse, they are not adequately implemented in practice.

2. ***Administrative support***

Unfriendly administrative support prevents children from disclosing abusive events. As a result, victimized children cannot obtain proper support, as explained by one of the NGO workers that participated in our study:

*“The country has hundreds of laws, but perpetrators are not punished as there is a lack of implementation. Sometimes, the offender is released from jail through bribe, absence of witnesses, social support, or power.”*

     -(KII-01, NGO Professional)

Many children are also unaware that they can get support from government hotline numbers. On the other hand, victims rarely complain to the police because the process in Bangladesh is not child friendly, as explained by an informant:

*“I was not aware of the government hotline numbers. As my family was not supportive while I was abused, I could not go to the local police station to seek support as I needed to be accompanied by a guardian.”*

     -(IDI-18, Grade 9)

3. ***Pedophilic behavior***

Pedophiles take pleasure in inflicting sexual abuse on children and some intentionally join child-based organizations to abuse children. Nonetheless, according to one of the interviewed NGO professionals, they often remain undetected in society:

*“Some pedophiles join child-based organizations with the intention of getting close to children easily. They collect all the information about children from the files and computers and target the vulnerable ones for abuse.”*

     -(KII-02, NGO Professional)

In some cases, pedophiles are relatives or very close friends of the child’s family. This implicit trust makes the children particularly vulnerable, as explained by one of our informants:

*“If a pedophile is a relative, it is difficult to protect the child. People usually rely on relatives to take care of their children. As parents do not know who a pedophile is, individuals they trust can abuse their children without being discovered.”*

     -(KII-02, NGO Professional)

In our cultural context, although this type of behavior is disrespectful to parents, they hide rather than disclose the abusive event.

4. ***Known perpetrator***

Our key informants concurred that perpetrators are usually known to the child, as being a family member, friend, or a relative gives them access to all the personal details they need to lure the victim into their trap. In addition, parents would never suspect a known person of abusing their children. One of the interviewed ICT teachers referred to the situation one of her students faced, as she was sexually abused online by her brother-in-law but could not avoid him because they lived in the same house:

*“A female student in grade 10 came to me with a complex situation that had been going on in her life. She lives with her sister for educational purposes. Her sister’s brother-in-law added her on Facebook and started asking her personal questions that offended her. All his conversations and gestures around the house indicate that he is interested in becoming physically involved, yet she feels that she cannot confide in anyone. She is afraid that if she told her sister, her sister’s marital life might be jeopardized. She cannot leave their house either because she has nowhere else to go. If she goes to the home village, she will be married off early and will not be able to continue her studies.”*

     -(KII-08, ICT Teacher)

5. ***Social stigma***

Children are raised with many societal stigmas, such as the internet being harmful and internet platforms being bad, among others. Yet, as they often use it secretly, they cannot ask their parents about the proper use of the internet, as explained by one of the respondents:

*“Bangladeshi children grow up in an environment promoting harmful social norms and traditions. This environment eventually contributes to online child sexual abuse.”*

     -(KII-06, ICT Teacher)

The cultural norms within families in Bangladesh encourage abused children to remain quiet, as the social structure in our country does not condone open discussions related to sexual events.

6. ***Availability of online sexual content***

Our informants noted that, as sexual content is widely available online and is shared freely in various social media groups, this increases the risk of OCSA. In most cases, perpetrators harass children by sending sexual material such as vulgar text messages, nude photos and videos on Facebook, Instagram, Snapchat, and other social media platforms, but children do not know how to stop such intrusive behavior. Some of the perpetrators also have access to the dark web, allowing them unsupervised access to a variety of sexual content. On this, a respondent stated:

*“People add me to many messenger groups that are full of sexual content. Even if I remove myself from one group, someone else adds me to another group. I do not want to join these groups intentionally.”*

     -(IDI-21, Grade 10)

As indicated above, in many cases, children automatically receive sexually explicit content from various online sources, as there is no strict barrier to such access. On this issue, one of the participating ICT teachers commented:

*“Many children complain to us (teachers) that they receive unwanted site links on the internet, which irritates them. There is no specific protection on the internet for children. Children are vulnerable and behave abnormally after seeing these contents unintentionally. Children who see pornographic videos suffer from mental disorders.”*

     -(KII-08, ICT Teacher)

7. ***Lack of outdoor games***

As open spaces for parks and playgrounds are scarce in cities, urban children do not get enough opportunities for outdoor games. Consequently, they are compelled to spend more time online for making friends, entertainment, and recreational activities, which increases their vulnerability to online abuse. According to a lawyer we interviewed:

*“Rural children have access to open spaces more than urban children. On the other hand, urban children have a wide availability of entertainment activities online, and they prefer to spend their time on the internet. However, even rural children want to make friends and spend time online because it has become more attractive than playing on the field.”*

     -(KII-05, Lawyer)

## Discussion

In this study, inadequate internet literacy has been identified as the root cause of online child sexual abuse (OCSA) in Bangladesh. While OCSA occurs in the virtual world, the existing social system plays a significant role in either increasing or reducing abuse ( Khondkar, 2021). Furthermore, we found that victims are unable to reveal their abusive events to those close to them due to social constraints. Social constraints are defined by society as unwritten patterns of behavior that adhere to social groups, shaping people’s behavior based on their sex and social conditions from an early age ( Johnstone, 2015; StudySmarter, 2022).

Based on the findings of this research, it has been determined that perpetrators engage in harassing children through various means, such as sending vulgar texts, nude photos, videos, and making online audio and video calls.

As most perpetrators hide their real identities during online interactions ( Arfini *et al.*, 2021) allows them to remain undetected. On the other hand, children do not feel free to disclose sexual events to their parents due to societal taboo surrounding such topics in Bangladesh. This issue is further exacerbated by the dismissive parenting strategies ( Deblinger and Runyon, 2005). Additionally, the UNODC (2020) highlights that many incidents of child sexual abuse go unreported to the police due to children’s fear of embarrassment in front of their friends, parents, and society.

We also found that the victims are reluctant to disclose their abusive experiences to legal authorities due to lack of a child-friendly legal system in Bangladesh.

This perspective is supported by the observations made by Warren (2019), indicating that the legal and administrative support for child sexual abuse fails to fully protect children, with no provision for anonymous reporting in Bangladesh. In fact, participants in this study identified the absence of anonymous reporting as a significant factor contributing to child sexual abuse.

The Government of Bangladesh has enacted several laws and regulations to combat and control OCSA, including the Prevention of Domestic Violence Act of 2010, the National Child Labor Elimination Policy of 2010, the National Children Policy of 2011, the Pornography Control Act of 2012, the Human Trafficking Deterrence and Suppression Act of 2012, and the Children Act of 2013. However, the improper implementation of these laws contributes to the growing prevalence of OCSA in our society ( Shamim, 2017).

Our findings further substantiated the prevailing notion that the child-parent relationship in Bangladesh is often strained. This issue, however, appears to be prevalent worldwide, as highlighted by Radford *et al.* (2015), who found that 50% of female children globally experience sexual abuse but never disclose it to anyone, and 7 out of 10 never seek help.

To address this growing social problem, the High Court of Bangladesh enacted legislation in 2022 requiring parents not to force their children into anything against their will in order to bring them up with positive attitudes ( The Daily Star, 2022). Nonetheless, the persistent gap in the parent-child relationship, as revealed in our study, renders children susceptible to OCSA.

Our findings revealed that if a child experience sexual abuse and tolerates it, there is a strong probability that the child will go on to abuse another child. This observation aligns with research from PsychCentral, which states that children who have been sexually abused often exhibit abnormal behaviors that can increase their vulnerability to further abuse or potentially lead them to become abusers themselves ( PsychCentral, 2022).

There is a common belief in Bangladeshi society that “good things happen to good people, and bad things happen to bad people” ( Gravelin *et al.*, 2019), which leads to victims being frequently blamed for the abuse they have endured. The theory of victim-blaming posits that victims bear partial or full responsibility for their own suffering, hardships, and other misfortunes ( Criminal Justice, 2022). Similarly, findings from the University of New Hampshire’s Crimes Against Children Research Center indicate that parents are more inclined to attribute blame to their children for the consequences of abuse, rather than holding the adults accountable ( Wright, 2012).

The study revealed that female children are the most frequent targets of online sexual abuse, yet parents tend to blame their daughters despite knowing that their children are not at fault. Additionally, our findings indicated that children endure severe psychological and physical consequences following online sexual abuse. According to the Council of Europe, young girls are especially vulnerable to sexual exploitation, abuse, and bullying by their peers in online environments. They may also exhibit physical symptoms and develop mental illnesses, such as a desire to isolate themselves and distance themselves from others ( Council of Europe, 2022).

Online child sexual abuse (OCSA) has emerged as a significant public health concern in Bangladesh, often stemming from romantic relationships with individuals met online ( Nova *et al.*, 2019). In fact, a longitudinal study conducted by Hébert *et al.* (2017) in Quebec revealed that 33% of the participating youth reported experiencing psychological violence within online romantic relationships. The authors further noted that victimized children encountered three times more psychological violence compared to non-victimized children. Our own findings align with this, as we discovered that children engaged in romantic relationships are more vulnerable to abusive situations. Perpetrators can exploit the private nature of chats by capturing screenshots, saving photos and videos, and later using them for online sexual abuse. The Australian government’s research findings support this, highlighting that children in romantic relationships often face image-based abuse, such as the circulation of nude photos or screenshots of sexually explicit content ( YLE, 2018). Additionally, children are frequently coerced into sharing nude photos and videos through threats.

Our analyses also indicate that relatives or close friends of the child’s family pose a particular risk, as the implicit trust in these individuals reduces the likelihood that pedophiles would be suspected of abuse. According to Tuli (2017), male pedophiles are more common than females in Bangladesh. On the other hand, in Europe, pedophiles tend to find their victims online. Yet, police investigations have also shown that pedophiles exist in many settings and even work at child-focused organizations ( YLE, 2018), supporting the findings obtained in the present study.

The study further revealed that Bangladeshi children are not well-equipped to use the internet safely. According to UNICEF (2019), children who engage in a various online activities require more developed internet skills, but children of low-income countries like Bangladesh do not have enough opportunities to gain proper knowledge of digital technologies. Moreover, Bangladeshi parents themselves lack knowledge about safe internet use, and are unaware of how perpetrators can exploit children via online platforms, they cannot protect their children. Although there are several parental control software tools available, parents are not adequately trained to utilize them ( UNICEF, 2019).

The similar findings revealed that children are abused due to a lack of understanding of internet use and the consequences of exploring the dark web ( Saprea, 2022). In an earlier study, Madden *et al.* (2006) proposed that children need more training on how to use the internet as a source of information that can keep them safe from being sexually abused online.

While agreeing with these observations, our study also revealed that excessive and unnecessary social media use contributes to OCSA. Children’s presence on various social media platforms such as TikTok, Facebook, Messenger, WhatsApp, Instagram, Snapchat, and others, may expose them to online sexual abuse by known and unknown individuals ( Kopecký and Szotkowski, 2018; Lovejoy, 2021). Although Facebook authorities have imposed some terms of service (ToS) restrictions on users under the age of 18, in practice children cannot be prevented from opening a Facebook account, as they can manipulate information to open an email ID ( Hargittai *et al.*, 2011). According to Kopecký and Szotkowski (2018), who conducted their study in Czech Republic, 66% of perpetrators use Facebook as a sexting platform. Similarly, O’Keeffe and Clarke-Pearson (2011) found that children are encouraged to engage in harmful activities by social media content such as movies, dramas, series, and so on, which influence children to have unusual relationships, drink alcohol, engage in sexual affairs, live together, and partake in a variety of inappropriate behaviors.

Using the internet has many benefits for children. Still, when children have easy access to internet-enabled devices like smartphones, tablets, laptops, etc., this encourages them to use the internet more often, thus increasing the risk of online sexual abuse ( Debroy, 2015). We also found that some children are addicted to watching pornography, and this may cause them to lose their moral values and suffer from a variety of health risks as well as mental disorders ( Baxter, 2014).

On the other hand, when 11- to 16-year-olds are prohibited from spending excessive time on their mobile phones, their academic scores improve ( Keeley and Little, 2017). Furthermore, as noted by our informants, insufficient space and safety for outdoor entertainment for urban children causes them to spend most of their free time online. This may cause a range of problems as children need physical activities that contribute to their mental and physical well-being, and develop skills such as group work, teamwork, and leadership qualities, but their outdoor activities have declined over the years due to safety concerns ( Kalpogianni, 2019). Similarly, it was found that the lack of outdoor entertainment is damaging for children’s health and well-being ( Bento and Dias, 2017).

We found that school dropouts and unemployed youths are more likely to become abusers because they spend most of their free time on the internet exploring unnecessary things. According to UNODC (2020) economic insolvency among youths, tend to develop bad attitudes and hang out with people who have bad traits, which in turn refrains them from developing good social norms and attitudes.

## Conclusion

The findings of this study pointed that the primary contributor to OCSA is inadequate internet literacy, posing an emerging issue and health concern for children in Bangladesh. Unfortunately, it has largely gone unexplored in Bangladesh until now, despite its significant impact on children’s physical and mental well-being. Further research and intervention programs are imperative to understand parental perceptions and enhance internet literacy among both children and parents concerning OCSA. The study highlights that children from both rural and urban areas face online sexual abuse. To protect children from such abuse, the government of Bangladesh should implement proactive measures. The implementation of internet literacy programs from secondary levels of education is crucial, as it will empower children with the knowledge to navigate online spaces safely. Moreover, there is a pressing need to strengthen law enforcement efforts and introduce amendments that establish an anonymous reporting system for children. By adopting these measures, the online platform can transform into a safer and more secure space for children in Bangladesh. The government, educators, and society at large should collaborate to safeguard the well-being of our children.

### Strengths

This qualitative study, investigating factors contributing to online child sexual abuse, is the first of its kind conducted in Bangladesh. The inclusion of participants from both urban and rural areas, as well as potential experts from relevant sectors, is a strength of this study.

While traditional interview studies carry a reliability risk by merely focusing on verbal meaning ( Finnegan, 2003), this study mitigates such risks by incorporating analysis of the latent meaning ( Krug *et al.*, 2002).

### Limitations

This study has several limitations that need to be acknowledged. Firstly, as the participants were children, there is a possibility of hidden abusive events or expressed unreal experiences due to the chance of disclosure to others, fear of humiliation and shame. Obtaining permission from the children’s parents reduced control over the first presentation of the research to the child, possibly exposing them to outside influences. Nova *et al.* (2019) explained that victims often remain silent about their abusive events due to being insulted or humiliated by their friends and family members. To encourage children to express themselves, we assure maintaining the privacy of all information, and only the research team members will have access to the data. Moreover, we assure them that their name will not be mentioned anywhere in the research documents.

Secondly, this study was conducted in two out of the 64 districts of Bangladesh, hence limiting the generalization of the findings. However, within the group of interviewees, we found data saturation, leaving us with no option to increase the sample size.

Thirdly, Bangladesh, being a densely populated country with 1265 people living in one square kilometer area ( Worldometers, 2023), poses difficulties in managing isolated places for interviews, especially in urban areas. Due to the overcrowded environment, conducting in-depth interviews (IDIs) in secluded locations was not always feasible. Furthermore, conducting interviews in separate places often sparked curiosity among family members, leading them to eavesdrop on the conversations, which disrupted the interview environment in some instances. However, our data collectors were properly trained to handle such situations by politely requesting the curious individuals to allow them 30 minutes of uninterrupted time with the interviewee. Additionally, to ensure cultural sensitivity, we engaged female data collectors for interviewing female children.

Fourthly, this study is one of few significant qualitative studies conducted in Bangladesh. Consequently, when supplementing the field data with input from relevant experts, there is a possibility of obtaining biased information. To address this risk, we took measures to include a diverse group of stakeholders, such as child psychiatrists, sociologists, child experts, teachers, and guardians.

Fifthly, the retrospective nature of the data in this study presents a potential challenge as the interviews focus on events from the children’s past, which may introduce recall bias. However, it is widely recognized that memories of events that evoke strong emotions tend to be well retained in the mind.

### Take-home messages

1. Online child sexual abuse (OCSA) is an emerging problem that has severe and detrimental effects on the physical and mental well-being of children. However, it remains relatively unexplored in Bangladesh.
2. The root cause of OCSA in Bangladesh is inadequate internet literacy, highlighting the urgent need for educational interventions.
3. The absence of laws to regulate OCSA in Bangladesh emphasizes the public health concern surrounding online child sexual abuse.

### Recommendations

The government of Bangladesh should include OCSA as a topic in secondary-level and higher-secondary-level education and should provide support for initiatives aimed at improving the internet literacy of the relevant stakeholders, including children, parents, teachers, and policymakers. The government should also take steps to make the overall internet space safe for children through scrutiny of all published content, including texts, images, and videos that children access. The government also needs to support outdoor sports and other activities for children. At the community level, awareness campaigns need to be initiated about safe internet use, mainly focusing on children and parents. Moreover, there should be a reporting system where children can anonymously report incidents of abuse. The media can play a vital role in reducing OCSA through mass awareness programs. The Children’s Act in Bangladesh needs to define and address OCSA and strict legal measures against the perpetrators of OCSA need to be ensured to protect children from abuse. Finally, additional studies are required to identify the most optimal intervention programs for improving children’s literacy regarding safe internet use.

## Data Availability

The data in this study is qualitative and contains sensitive information. During the data collection process, we assured respondents that their information would not be openly disclosed and that we would only share this data for research purposes. Accordingly, access to these data can be granted upon request to the corresponding author, Professor Md Atiqul Haque, at < atiqulm26@bsmmu.edu.bd>. The data will be provided upon reasonable request, specifically for social science academics and researchers, to further the betterment of society. Mendeley data and DANS Data: These files contain the qualitative semi-structured guideline, https://easy.dans.knaw.nl/ui/datasets/id/easy-dataset:321437 ( Islam, 2023) used for conducting in-depth interviews and key informant interviews to explore online child sexual abuse in Bangladesh. The original study was a mixed-method study. This file also contains the “Consolidated criteria for reporting qualitative studies (COREQ)” and the “SAGER guidelines for Sex and Gender Equity in research” for this study, https://data.mendeley.com/datasets/8y7s8wwkcy/1 ( Salwa and Haque, 2023).
