# Supplementary material for: Factors contributing to online child sexual abuse in Bangladesh: A qualitative inquiry
Source: F1000Res. 2024 Aug 9;12:1364. Originally published 2023 Oct 18. [Version 3] doi: 10.12688/f1000research.141568.3 (PMC11143399; doi:10.12688/f1000research.141568.3)
Supplement: Peer review history

# Reviewer report: Reviewer response for version 2

**DOI:** 10.5256/f1000research.161302.r238287

**Published:** 2024-05-31

**Authors:** Salim Mahmud Chowdhury

**Affiliations:** 1 Centre for Injury Prevention and Research Bangladesh, Dhaka, Bangladesh

All comments are well addressed. I don't have any further comments. This article can be indexed without any reservation.

Is the work clearly and accurately presented and does it cite the current literature?

Yes

If applicable, is the statistical analysis and its interpretation appropriate?

Not applicable

Are all the source data underlying the results available to ensure full reproducibility?

Yes

Is the study design appropriate and is the work technically sound?

Partly

Are the conclusions drawn adequately supported by the results?

Yes

Are sufficient details of methods and analysis provided to allow replication by others?

Yes

Reviewer Expertise:

Health system research and injury prevention

I confirm that I have read this submission and believe that I have an appropriate level of expertise to confirm that it is of an acceptable scientific standard.

# Reviewer report: Reviewer response for version 2

**DOI:** 10.5256/f1000research.161302.r239820

**Published:** 2024-02-09

**Authors:** N. M. Rabiul Awal Chowdhury

**Affiliations:** 1 Department of Anthropology, Comilla University, Cumilla, Chittagong Division, Bangladesh

Number of IDIs and KIIs are mentioned but how these number are justified is not clarified. Any other relevant socio-cultural issues, except rural-urban, may influence in selecting the participants of the study like, gender, religion, financial condition etc. which should consider in selecting the respondents purposively. 

 Gender is a significant social phenomenon which may play its imperative role in such topic but findings are not representing any difference and variation for boys and girls on this study topic, which drawn my attention. 

 A diagram of mentioned conceptual framework of this study would manifest easy and better understanding 

 More compiled and integrated (IDIs and KIIs) interpretation of each categories and sub-categories of findings would enrich the result section rather too many statements of the respondents

Is the work clearly and accurately presented and does it cite the current literature?

Yes

If applicable, is the statistical analysis and its interpretation appropriate?

Not applicable

Are all the source data underlying the results available to ensure full reproducibility?

Yes

Is the study design appropriate and is the work technically sound?

Partly

Are the conclusions drawn adequately supported by the results?

Yes

Are sufficient details of methods and analysis provided to allow replication by others?

Partly

Reviewer Expertise:

health system, NCDs, SRHR,

I confirm that I have read this submission and believe that I have an appropriate level of expertise to confirm that it is of an acceptable scientific standard.

# Response

**Published:** 2024-08-03

**Authors:** Md Redwanul Islam

**Affiliations:** Public Health and Informatics, Bangabandhu Sheikh Mujib Medical University, Dhaka, Bangladesh

**Reviewer point #1:** Number of IDIs and KIIs are mentioned but how these number are justified is not clarified. Any other relevant socio-cultural issues, except rural-urban, may influence in selecting the participants of the study like, gender, religion, financial condition etc. which should consider in selecting the respondents purposively.

**Author response #1:** Thank you for your suggestions. We have addressed all the comments you suggested in the latest version of the manuscript. We have clarified the justification for including the number of Key Informant Interviews (KIIs) and In-Depth Interviews (IDIs) in the 'Study Procedure' section under the sub-section of 'Methods'. We included KIIs from subject-specific stakeholders from multiple disciplines, such as child psychiatrists, ICT teachers, child rights activists, and psychologists. IDIs included students from rural and urban perspectives, with equal participation from both males and females. Data were collected until saturation was reached.

**Reviewer point #2:** Gender is a significant social phenomenon which may play its imperative role in such topic but findings are not representing any difference and variation for boys and girls on this study topic, which drawn my attention.

**Author response #2:** We did not analyze the study findings separately for boys and girls, but this could be a recommendation for further study. To our knowledge, this type of qualitative assessment is unique in Bangladesh, that is why we identified the root cause but did not identify the separate roles.

**Reviewer point #3:** A diagram of mentioned conceptual framework of this study would manifest easy and better understanding

**Author response #3:** We have made the conceptual framework easier and better to understand by using a Figure.

**Reviewer point #4:** More compiled and integrated (IDIs and KIIs) interpretation of each categories and sub-categories of findings would enrich the result section rather too many statements of the respondents

**Author response #4:** We have compiled the interpretations in the discussion section. As this phenomenon is new from a country perspective, we wish to include more statements for a better understanding of the phenomenon. Thank you once again for your time and consideration.

# Reviewer report: Reviewer response for version 1

**DOI:** 10.5256/f1000research.155026.r216676

**Published:** 2023-11-29

**Authors:** Salim Mahmud Chowdhury

**Affiliations:** 1 Centre for Injury Prevention and Research Bangladesh, Dhaka, Bangladesh

Language editing is needed. Conclusion section of the abstract has study findings (better to focus on policy implication of the study). Rural urban population distribution mentioned in the introduction section needs to be reviewed. Operational definition of youth is needed. Please check the strength and limitations of study section to avoid repetition of same information. Conclusion section should be more structured to make this paper reader-friendly.

Is the work clearly and accurately presented and does it cite the current literature?

Yes

If applicable, is the statistical analysis and its interpretation appropriate?

Not applicable

Are all the source data underlying the results available to ensure full reproducibility?

Yes

Is the study design appropriate and is the work technically sound?

Partly

Are the conclusions drawn adequately supported by the results?

Yes

Are sufficient details of methods and analysis provided to allow replication by others?

Yes

Reviewer Expertise:

Health system research and injury prevention

I confirm that I have read this submission and believe that I have an appropriate level of expertise to confirm that it is of an acceptable scientific standard, however I have significant reservations, as outlined above.

# Response

**Published:** 2024-01-11

**Authors:** Md Redwanul Islam

**Affiliations:** Public Health and Informatics, Bangabandhu Sheikh Mujib Medical University, Dhaka, Bangladesh

Thank you for your suggestion. We agreed with your feedback and addressed all the comments in the manuscript with changes marked. We have enhanced the language throughout the manuscript. The conclusion section of the abstract has incorporated the policy implications of the study. In the introduction section, we have revised the distribution of the rural-urban population. We agreed with the reviewer and provided an operational definition for youth. The strengths and limitations section has been reviewed to avoid repetition of information. The conclusion section has been modified to make it reader-friendly.
